# The impact of physical activity on mental health across diverse populations in Saudi Arabia: a narrative review

**DOI:** 10.3389/fpsyg.2026.1795785

**Published:** 2026-08-07

**Authors:** Amani Alharthy

**Affiliations:** Department of Health Science, College of Health and Rehabilitation Sciences, Princess Nourah bint Abdulrahman University, Riyadh, Saudi Arabia

**Keywords:** mental health, narrative review, physical activity, Saudi Arabia, young adults

## Abstract

The current literature review presents information about the association of PA in connection with the results of mental health from the viewpoint of studies conducted among various samples such as university students, teenagers, and healthy people. This narrative review examines the interrelationships between physical activity (PA), mental health, and the sociocultural environment among diverse populations in Saudi Arabia. A comprehensive literature search was conducted across PubMed, Scopus, Web of Science, Embase, and Google Scholar for studies published through 2025. The findings of the review reveal that PA also positively affects sleep, cognitive functioning, and quality of life. Finally, intervention research reveals a positive effect of PA on emotional, social, and motivational functioning despite the small number of high-quality studies. The factors preventing PA are diverse and may be considered under the umbrella of social (i.e., a lack of peer and family support), environmental (i.e., an unattractive and unfriendly environment) and cultural (i.e., restrictive cultural norms affecting the accessibility of women to sporting facilities) barriers. Although national interventions under the Saudi Vision 2030 program would encourage participation through raising awareness, constructing gyms, and creating a conducive urban environment for this purpose, the current research does not provide insights into the impact of the proposed interventions on the stated outcome variable. There is a need for longitudinal research that would investigate the impact of interventions on different groups (i.e., non-students). Also, no gender-sensitive interventions have been tested so far. The impact of national policy should be studied further as well.

## 1 Introduction

Mental health among young adults is a new public priority in Saudi Arabia. It is a relatively new issue of concern in the country of Saudi Arabia as far as young adulthood is concerned. According to research findings, depression, anxiety, and stress disorders have been identified as endemic diseases in this population caused by both academic issues, lifestyle challenges, and fast-paced cultural transformation processes (AlFaris et al., 2016; Qureshi et al., 2013). Young adults who are either university students or working adults suffer from the effects of two important phases simultaneously: adjusting to a new environment as well as facing societal pressures of growing into an adult. Ignoring this issue could lead to various negative consequences.

In addition, inactivity among the population of Saudi Arabia has become another widespread phenomenon, and this can be regarded as an important public health problem as well. Research data indicate that more than 70 percent of adult Saudis do not follow the recommendations provided by the WHO concerning regular physical activities (Al-Hazzaa, 2018). Besides contributing to obesity and cardiovascular diseases, inactivity among the local population negatively influences their psychological state. Changes in lifestyles that include greater utilization of automobiles and technologies, along with a sedentary approach toward leisure, have resulted in reduced levels of physical activity (Al-Hazzaa, 2018; Alsubaie and Omer, 2015; Abdelhay et al., 2025). However, adverse climatic conditions and the lack of walkable environments prevent physical activity among Saudi adults. Cultural factors primarily influence women (Al-Hazzaa, 2018; Alsubaie and Omer, 2015; Abdelhay et al., 2025).

Physically active lifestyles have become popular globally due to the fact that it is an affordable and scalable intervention aimed at improving psychological health (Ruiz-Ranz and Asín-Izquierdo, 2025). Globally, evidence suggests that physically active individuals tend to have fewer depression, anxiety, and stress-related symptoms along with greater resilience, higher-quality sleep, and better cognitive functions (Haapala et al., 2025; Yang and Zikos, 2025; Wiklund et al., 2025). As opposed to pharmacological interventions, physical activity does not produce any side effects and can be easily implemented into the daily routine of patients. It should be noted that physical activity in the context of Saudi Arabia is a very effective way to cope with mental issues due to a small number of mental health specialists in relation to the population.

The significance of this topic is heightened by broader socio-emotional transformation currently underway in Saudi Arabia. Vision 2030, the Kingdom’s ambitious reform initiative, seeks to increase quality of life, healthier living, and free space for recreation and sport (Burger and Arampatzi, 2025). Higher levels of physical activity are more than just an issue of health; it is also a culture and economic concern which is addressed through policies encouraging community sports, participation of females in sport and urban development strategies encouraging active lifestyles. Insight into the connection between physical activity and mental health in Saudi youth, therefore, has practical implications as well and will allow the utilization of evidence for informing healthcare policy and social policy.

Though much has been done to understand and address this issue, little evidence exists regarding PA and mental health in Saudi Arabia. Available literature on the issue is cross-sectional in design and, most often, focuses on college/university students. Moreover, none of the reviewed studies controlled for cultural and gender factors. Therefore, there is a necessity to undertake a narrative synthesis of available information to inform policy, practice, and further research in the area. This review seeks to shed light on the interrelationships between physical activity, mental health, and sociocultural environment of Saudi youth. The Saudi sociocultural context in the present research study is defined as a distinct interrelation of family values, gender norms, and social determinants affecting the access to recreational activities. Lastly, encouragement of physical activity might be the most realistic and efficient strategy to reduce the burden of mental health illnesses in Saudi youth.

## 2 Materials and methods

For evaluating the association between physical activity and mental well-being in Saudi Arabia, a comprehensive search strategy was implemented. Databases utilized for performing the search included PubMed, Scopus, Web of Science, Embase, and Google Scholar until literature published up to 2025 was included. The literature search utilized standardized keywords including ‘physical activity,’ ‘exercise,’ ‘mental health,’ ‘depression,’ ‘anxiety,’ ‘stress,’ ‘quality of life,’ and ‘Saudi Arabia,’ along with relevant geographical variations. The search was executed using the following string: (‘physical activity’ OR ‘exercise’) AND (‘mental health’ OR ‘depression’ OR ‘anxiety’ OR ‘stress’ OR ‘quality of life’) AND (‘Saudi Arabia’). Search inclusion criteria involved (1) population studies in Saudi Arabia, (2) healthy adolescents and young adults, (3) physical activity and mental well-being or quality of life, (4) articles written in English. The search exclusion criteria comprised the following: (1) Studies conducted outside of Saudi Arabia, (2) non-empirical studies, (3) individuals outside the target age range of 12 to 35 years, and (4) Populations that suffered from serious mental disorders.

This review specifically examines the link between PA levels and mental well-being outcomes. Quality of life measures were included as they are robust proxies for mental well-being in the literature. Data were synthesized using a narrative approach to map trends across adolescents and young adults, with particular attention to sociocultural barriers.

## 3 Study results

### 3.1 Characteristics of included studies

The characteristics of the included studies are summarized in Table 1. Most studies were conducted in Saudi Arabia, including national surveys and regional studies from cities such as Riyadh, Jeddah, Makkah, and Jazan, with one study conducted in the United Arab Emirates. Sample sizes varied considerably, ranging from 27 participants in an intervention study to national surveys including over 26,000 families and systematic reviews encompassing up to 29,580 participants. The review included diverse populations, including children, adolescents, university and medical students, young adults, community-dwelling adults, female university students, individuals with disabilities, and national population samples. The evidence comprised predominantly cross-sectional studies, along with intervention studies and systematic reviews, providing broad representation across different age groups, settings, and participant characteristics.

**Table 1:** Characteristics of studies included in the review.

Author/year	Study design	Sample characteristics	Outcomes assessed	Key findings
Abou Abbas and AlBuhairan (2017)	National cross-sectional survey	Adolescents 10–19 yrs.; *n* = 12,121; mean age: 15.7 ± 3.4 years;	Emotional symptoms (sadness, hopelessness, worry)	High emotional distress, especially females/older adolescents; linked to poor parent relationships, negative body image, chronic illness
Al-Eisa and Al-Sobayel (2012)	Cross-sectional	Female students, *n* = 105; mean age: 26.3 ± 7.1 years	PA, depression	Higher PA associated with lower depression; sedentary behavior increased risk
Al-Eisa et al. (2014)	Cross-sectional	Female students, *n* = 76; mean age 20.9 ± 1.4 years	PA, academic stress	PA reduced academic stress; sedentary lifestyle worsened stress
Al Balawi et al. (2019)	PHC-based cross-sectional study	Young adults, *n* = 384	Depression (PHQ-9)	Depression prevalence 74%; associated with female sex, marital/financial problems, poor social support, sleep disorders
Alfakeh et al. (2024)	Cross-sectional	Youth, *n* = 2,787; mean age: 20.37 ± 2.2 years	Depression, stress, psychological distress	5.1% extreme depression; 28.6% distress; female sex and regional variation significant; undiagnosed depression linked to higher scores
Alhabeeb et al. (2022)	Cross-sectional	Adults, *n* = 6,015; mean age: 36.45 ± 12.45 years	MDD, GAD risk	MDD: 12.7%; GAD: 12.4%; smoking (incl. waterpipe) increased risk; PA and screening protective; female sex, low income/education increased risk
Al-Nuaim et al. (2012)	National adolescent survey	Adolescents 15–19 yrs., *n* = 1,270; mean age: 17.08 ± 1.27 years	PA patterns	Males more active; rural desert least active; PA lower in females, obese, older adolescents; regional/school-type differences
Abdulrashid et al. (2023)	Cross-sectional	Young adults; *n* = 1,200	PA, depression	PA protective against depression; inactive individuals had higher depressive symptoms
AlHamad and AlAmri (2021)	Community cross-sectional study	Adults ≥13 yrs., *n* = 467; mean age: 27.0 ± 10.9 years	Depression (PHQ-9)	Mild–severe depression common; associated with female sex, excessive social media use, high negative affect
Aljehani et al. (2022)	Mixed-methods university study	Female university students; *n* = 375; mean age: 19.9 ± 1.7 years	PA levels; barriers/facilitators	70% below WHO PA cutoffs; barriers: cultural norms, workload, lack of facilities; facilitators: family support, health awareness
Almaged et al. (2021)	Cross-sectional	University students; *n* = 500;	PA, anxiety, depression	PA inversely associated with anxiety/depression; sedentary time harmful
Alnofaiey et al. (2023)	Cross-sectional	*n* = 2,819; mean age: 22.17 ± 2.28 years	PA, sleep quality, depression	PA improved sleep quality and reduced depressive symptoms
Alqahtani et al. (2021)	National survey	26,000 families	PA levels (≥150 min/week)	Only 17.4% met PA guidelines; nationally low PA levels; urgent need for interventions
Alsagah et al. (2021)	Systematic review (18 studies)	College/University Students (Adolescents)	PA prevalence	PA 4–44.5%; widespread inactivity; high fast-food intake, low fruit/veg intake
Alshehri et al. (2020)	School-based cross-sectional study	Adolescents 12–16 yrs., *n* = 451; mean age: 14.87 ± 0.94 years.	General MH problems (SDQ)	23.5% likely MH problems; linked to non-authoritative parenting, insecure attachment, weak social support
Alsulami et al. (2023)	Cross-sectional obesity/PA study	Adults in Makkah, *n* = 2,115; mean age: 33.52 ± 13.12	PA behaviors, obesity	Obese individuals less active; PA protective against obesity
AlTamimi et al. (2022)	Cross-sectional study	Young men 20–35 yrs., *n* = 3,600	PA patterns, inactivity	24.9% inactive; Saudis more inactive than Filipinos; inactivity linked to age, residency, income, education, BMI
Altwaijri et al. (2023)	National mental health household survey	Youth 15–30 yrs., *n* = 1,881	Anxiety, disruptive behavior, mood, eating, substance disorders	Any disorder: 40%; anxiety most common; female sex, low education, family history increased risk; treatment uptake only 14%
Amr et al. (2013)	University cross-sectional study	University students, *n* = 1,696; mean age: 20.9 ± 1.9 years	Depression, anxiety, suicidal ideation	Depression 24.4%; GAD 14%; suicidal ideation 1.1%; linked to female sex, college type, financial/personal problems
Bashatah et al. (2023)	Community survey	Adults in Riyadh, *n* = 354	PA, sedentary time	63% reported weekly PA; sedentary time 9.5 h/day; walking most common; PA associated with education, employment, income
Beyari (2023)	Analytical Hierarchical Process	Youth, *n* = 385	Social-media–linked MH decline	Validation-based features (likes, comments, followers) most harmful; entertainment features less harmful
Gmmash et al. (2023)	Cross-sectional	Adults, *n* = 27; mean age: 14 ± 2.38 years,	PA, mental wellbeing	PA strongly associated with better wellbeing; inactivity linked to poor mental health
Kokandi et al. (2019)	Cross-sectional	University students, *n* = 1,026	PA, stress	PA inversely associated with stress; females reported higher stress
Mahfouz et al. (2023)	School-based observational cross-sectional survey	Students in Jazan, *n* = 601	Depression, anxiety, stress, QoL (DASS-21, PedsQL)	PA positively correlated with QoL; reduced depression/anxiety/stress post-COVID return to school
Nour et al. (2023)	Systematic review (46 studies)	Adults, *n* = 25,814	Depression	Pooled prevalence 37.3%; risk factors: female sex, single status, low education, poor housing, medical illness, smartphone addiction
Nour et al. (2023)	Systematic review (44 studies)	Saudi college/university students, *n* = 25,814	PA levels	Only 27% met WHO cutoffs; barriers: time, motivation, resources; facilitators: health, social support, weight management
Said and Shaab (2022)	School-based cross-sectional study	Students 10–15 yrs., *n* = 981; mean age: 13.41 ± 1.17 years	PA, sedentary behavior, BMI	Boys more active; nearly all used cars/buses; BMI linked to PA, sedentary behavior, breakfast habits
Zahra et al. (2022)	Cross-sectional	Adolescents, *n* = 359	PA, QoL, mental wellbeing	PA positively associated with QoL and wellbeing; low PA linked to poorer mental health

### 3.2 Mental health trends among Saudi youth

Surveys of the general population and universities continue to identify an alarmingly high rate of psychological problems among Saudi youth. According to SNMHS, along with the epidemiological evidence provided by SNMHS, the majority of mental health conditions manifest during the late adolescent and early adult periods, which are associated with significant life transitions and susceptibility (2). University students seem highly vulnerable, since almost half of the students enrolled in medical and health science courses display depressive symptoms, due to academic stress, lack of sleep, and social isolation (1). Based on the analysis of the scholarly literature, it is evident that young Saudi people experience similar issues associated with the complexity of their problems, which are represented by the high frequency of mental illnesses, along with various factors affecting the likelihood of developing such disorders (see Table 2). This trend is supported by data obtained during the review of the selected studies. A nationwide survey involving more than 12,000 participants indicated that feeling sad, hopeless, and worried is quite frequent, with the emotional states intensifying among females and older participants (Abou Abbas and AlBuhairan, 2017). Negative parent–child interactions, poor body image, and the presence of chronic disease were considered as predictors of higher probability to feel negative emotional states (Abou Abbas and AlBuhairan, 2017). Thus, it could be concluded that familial and somatic aspects are vital factors affecting the mental health of people. Moreover, the sub-sample of Saudi National Mental Health Survey in the age group from 15 to 30 confirmed the severity of the problems (Altwaijri et al., 2023). The life-time prevalence of any disorder was found to be 40%, including the following disorder types: anxiety (26.8%), disruptive behavior (15.4%), mood (9.7%), eating (7%), and substance use disorders (4%) (Altwaijri et al., 2023). Although the numbers were impressive, less than 14% of all treatable cases received the appropriate treatment; thus, treatment issues were found along with barriers to receiving mental health care (Altwaijri et al., 2023).

**Table 2:** Common mental health disorders among young adults in Saudi Arabia with associated risk/protective factors.

Author/year	Population and sample size	Common disorders/symptoms	Prevalence	Key risk/protective factors
Abou Abbas and AlBuhairan (2017)	Adolescents 10–19, *n* = 12,121	Sadness, hopelessness, worry	High prevalence, esp. females and older adolescents	Poor parent relationships, negative body image, chronic illness
Altwaijri et al. (2023)	Youth 15–30, *n* = 1,881	Anxiety, disruptive behavior, mood, eating, substance disorders	Any disorder: 40%; Anxiety: 26.8%; Mood: 9.7%	Female sex, lower education, family history, low treatment uptake (14%)
Al Balawi et al. (2019)	Young adults 20–40, *n* = 384 (PHC, Tabuk)	Depression	74% (38% mild, 21% moderate, 15% severe)	Female sex, marital/financial problems, poor social support, sleep disorders
Alshehri et al. (2020)	Adolescents 12–16, *n* = 451	General MH problems (SDQ)	23.5% likely MH problems	Lack of authoritative parenting, insecure attachment, weak social support
Nour et al. (2023)	Adults, systematic review (46 studies; *n* = 25,814)	Depression	37.3% pooled prevalence	Female sex, being single, low education, poor housing, medical illness, smartphone addiction
AlHamad and AlAmri (2021)	Adults ≥13 yrs., *n* = 467 (Riyadh)	Depression (PHQ-9)	34.3% mild, 24.2% moderate, 10.1% moderately severe, 5.8% severe	Female sex, excessive social media use, high negative affect
Amr et al. (2013)	Univ. students, *n* = 1,696 (KFU)	Depression, anxiety	24.4% depression; 14% GAD; 1.1% suicidal ideation	Female sex, type of college, financial & personal problems
Alhabeeb et al. (2022)	Adults, *n* = 6,015 (national screening)	MDD, GAD risk	MDD: 12.7%; GAD: 12.4%; diagnosis rates <2%	Female sex, low income/education, smoking, waterpipe use; Protective: PA, hobbies, volunteering
Alfakeh et al. (2024)	Youth, *n* = 2,787	Depression, stress, psychological distress	40% normal; 5.1% extreme depression; 28.6% distress	Female sex, regional variation, undiagnosed depression linked to higher scores
Beyari (2023)	Youth, *n* = 385	Mental health decline linked to social media features	Not quantified; strongest with validation-based features	Likes/comments/followers biggest contributors; entertainment less harmful

A study conducted among young adults who visited primary care clinics in Tabuk reported a high prevalence rate of depression at 74%, and the majority of those affected had mild to moderate depression, while there were also many cases of severe depression (Al Balawi et al., 2019). Social determinants were highly significant risk factors such as lack of social support, marital conflicts, money worries, stress in life events, history of depression, and sleep disturbances (Al Balawi et al., 2019). Significantly, 10 percent of these patients had contemplated suicide, which indicates that there is an urgent need for prevention programs (Al Balawi et al., 2019). In addition to individual risk factors, social factors such as family factors also played a role in affecting adolescent mental health. In disadvantaged neighborhoods, 23.5 percent of Saudi adolescents were expected to develop mental illness (Alshehri et al., 2020). The protective factors were authoritative parenting, secure attachment, and strong social support, while the absence of these protective factors was a risk factor (Alshehri et al., 2020). It is evident that family and community prevention programs are crucial.

However, a newer study through meta-analysis and systematic review including 46 studies based on more than 25,000 Saudi adults showed a prevalence rate of 37% for depression (Nour et al., 2023). Moreover, subgroup analysis revealed the existence of high rates of depression for females, single individuals, and university students, along with the presence of risk factors that included poor educational attainment, poverty, illness conditions, insomnia, and smartphone addiction (Nour et al., 2023). These national estimates show similar trends as youth studies on mental health issues and indicate widespread occurrence of these problems.

Besides conventional academic and social pressures, cyber life is now becoming an influencing factor on the psychological well-being of Saudi youth. The association between social media consumption and depression has been discussed in many research studies. As early as 2020, AlHamad and AlAmri (2021) reported a study conducted in which almost 40% of the adult population in Riyadh that excessively consumed SNS suffered from moderate to severe depression. The gender differences were evident as the prevalence rate for women regarding moderate to severe depression was relatively much higher compared to males (AlHamad and AlAmri, 2021). It should be noted that depressive symptoms had a significant positive association with the extent of SNS consumption; moreover, social media consumers reported higher feelings of negativity including anger and dissatisfaction (AlHamad and AlAmri, 2021).

For example, Beyari (2023) scrutinized the nuanced influence of some social media features on teen mental health (Beyari, 2023). The study found that features encouraging social validation (such as likes, comments, followers) had the strongest association with negative mental health outcomes, while entertainment and information exchange features had comparatively lesser correlations (Beyari, 2023). These observations suggest that the ways in which adolescents interact with online spaces, rather than usage levels, explain substantial mental health threats. The issue of high rates of depression and anxiety continues to persist among university students. In King Faisal University students, almost 25% reported depressive symptoms, 9.9% being classified to have major depression (Amr et al., 2013). Financial concerns, personal troubles, and female sex were all significant predictors, supporting previous associations with gender and socioeconomic stressors (Amr et al., 2013). Such results reflect those of more contemporary national and regional prevalence studies which confirm that women are at much greater risk than men of experiencing mental disorders (Alhabeeb et al., 2022). There is information regarding prevalence obtained through national surveys which might give us an idea of the magnitude of the problem. According to a 2022 study of over 6,000 Saudis, 12.7% of people were at risk for developing MDD while 12.4% had GAD (Alhabeeb et al., 2022). Nonetheless, the diagnosis and treatment rates were extremely low standing at 1.5 and 0.5% correspondingly (Alhabeeb et al., 2022). The associated risk factors included being a woman, poor education, low socioeconomic status, as well as engaging in risky behavior such as smoking and smoking with water pipe (Alhabeeb et al., 2022). On the other hand, physical activity, volunteer work, and leisure proved to be protective factors (Alhabeeb et al., 2022).

The most recent youth study in the country did a survey on more than 2,800 Saudi youths and reported more than a quarter of participants exhibited psychological distress at a very high level (Alfakeh et al., 2024). While about 40% reported average levels of depression, 5.1% had severe depression, and similar findings were observed across genders and geographical locations (Alfakeh et al., 2024). Importantly, individuals reporting symptoms of depression without receiving diagnoses of such symptoms recorded higher depression scores, indicating the existence of underdiagnosis of mental disorders. In sum, these studies provide strong evidence that adolescents and youths in the Kingdom of Saudi Arabia are exposed to combination of conventional and contemporary sources of stresses (scholarship, financial, family pressures, overexposure to digital technologies, and lifestyles, respectively). Gender disparities across the various studies conducted in the country underscore the importance of examining this variable further. More importantly, information on the protective role played by physical exercise and other leisure activities in these national surveys naturally leads to the next section of this paper.

### 3.3 Physical activity levels among young adults in Saudi Arabia

PA remains an intractable public health issue in Saudi Arabia, especially among youth and young adults. Many researchers emphasize the interplay between environment, culture, and behavior, all of which restrict possibilities for prolonged physical activities (Table 3). Dependence on vehicles for travel, adverse weather conditions, and lack of easy access to fitness centers remain impediments to unrestricted participation in physical activities (Al-Hazzaa, 2018; Al-Zalabani et al., 2015). This is more pronounced among women due to their having to contend with issues of culture, lack of gender-exclusive facilities, and clashing responsibilities intellectually and domestically (Alajlan et al., 2024).

**Table 3:** Studies on physical activity levels among Saudi youth and young adults.

Author/year	Population and sample size	Prevalence/patterns of PA	Key findings (barriers/facilitators/associations)
Alqahtani et al. (2021)	National survey, *n* = 26,000 families	17.4% met ≥150 min/week	Nationally low PA levels; urgent need for interventions
AlTamimi et al. (2022)	Young men (20–35 yrs), *n* = 3,600	24.9% inactive overall; 41.5% Saudis vs. 14% Filipinos	Inactivity linked to age, residency, higher income, education, BMI
Alsulami et al. (2023)	Adults in Makkah, *n* = 2,115	Obesity: 23%; Overweight: 32.8%	Obese less active (walking, jogging, strength); PA protective against obesity
Alasqah et al. (2021)	Adolescents, systematic review (18 studies)	PA prevalence 4–44.5%	High fast-food consumption, low fruit/veg intake, widespread inactivity
Said and Shaab (2022)	Students 10–15 yrs., *n* = 981	Boys more active; nearly all used cars/buses	BMI linked to PA, sedentary behaviors, breakfast habits
Aljehani et al. (2022)	Female university students, *n* = 375 (+interviews)	70% below WHO PA cutoffs	Barriers: cultural norms, workload, lack of facilities; Facilitators: family support, health awareness
Nour et al. (2023)	46 studies, Saudi college students, *n* = 25,814	Only 27% met WHO cutoffs	Barriers: time, motivation, resources; Facilitators: health, social support, weight management
Bashatah et al. (2023)	Adults, Riyadh region, *n* = 354	63% reported some weekly PA; avg. 9.5 h/day sedentary	Walking most common; sedentary time higher among women; PA associated with education, employment, income
Al-Nuaim et al. (2012)	Adolescents 15–19 yrs., *n* = 1,270	Males more active; rural desert least active	PA lower in females, obese, and older adolescents; regional and school-type differences

According to national survey statistics, merely 17.4% of adult Saudis adhere to the WHO recommendations regarding their PA levels, leaving over 80% physically inactive (Alqahtani et al., 2021). Indeed, recently, a community survey in Riyadh revealed similar results and identified that physical inactivity is not an issue of choice but a result of other more complex factors. Insufficient motivation and distractions due to studies or work were listed as the primary causes for this trend (Alhwoaimel et al., 2025). As expected, further analysis demonstrates similar trends because of their sociodemographic character. Thus, AlTamimi et al. (2022) concluded that 41.5% of Saudi males reported physical inactivity in comparison with 14% of Filipinos. Therefore, the cultural differences between these ethnicities, living within one country, should be taken into account (AlTamimi et al., 2022). The following variables positively correlated with physical inactivity among Saudis: increasing age, income, education level, and BMI (AlTamimi et al., 2022). In addition, Alsulami et al. (2023) confirmed positive connections between PA level and obesity, showing that obese people have fewer jogging or strength training days than lean ones (Alsulami et al., 2023). Moreover, obesogenic risk factors demonstrated a negative correlation with daily walks and moderate activities.

In adolescents, for instance, Alasqah et al. (2021) have pointed out dangerously low levels of PA from 4 to 44.5%, in addition to prevalent unhealthy eating habits, such as junk food and skipping breakfast (Alasqah et al., 2021). Such behaviors at a young age are the most probable factors to contribute to the rise in obesity rates and related diseases. Similarly, Said and Shaab (2022) have shown that the aforementioned variables were significantly influential on BMI among 10–15-year-old individuals, and almost universal reliance on cars and buses was yet another aggravating factor.

For university students, who are in a vital stage of development for adopting healthy practices for life, there are some serious issues of concern. According to a study conducted by Aljehani et al. (2022), 70% of the female students do not comply with the recommendation of WHO on moderate physical activity and 62% of them do not comply with the guideline related to vigorous activity (Aljehani et al., 2022). The facilitators in their case were family encouragement and awareness about the benefits of health while the barriers included academic pressures, lack of facilities, and culture-related restrictions. A systematic review of 44 studies also pointed out that only 27% of Saudi University students comply with WHO guideline on physical activity.

The information gained from cross-sectional questionnaires in Riyadh is also useful: while 63% of young adults participated in some form of PA on a weekly basis, there was still much more inactivity, and people sat for an average of 9.5 h per day (Bashatah et al., 2023). Walking was found to be the most popular activity, along with bodybuilding; however, females sat more often than men. Employment and education levels played a significant role in terms of physical activity; thus, once again, the importance of socioeconomic factors should be considered. Regional disparities have been reported as well. For instance, Al-Nuaim et al. (2012) reported that adolescents who resided in the rural desert were less physically active than those in the cities, and boys always exhibited more physical activity than girls regardless of age (Al-Nuaim et al., 2012). Overall, these studies indicate a fairly unified picture of very low PA participation levels among Saudis, due to a variety of reasons including obesogenic environment, culture, and structure. However, there is clear evidence that specific interventions can make a significant difference and result in high PA levels. These include making places available for physical activity safe, encouraging active transportation, and designing specific programs for women and students.

### 3.4 Evidence linking physical activity and mental health

The studies conducted in Saudi Arabia have become progressively evident regarding the positive association between PA and mental wellbeing, yet, at the same time, there is still dispersion of evidence based on ages, samples and outcomes (Table 3). The cross-sectional and intervention studies help reveal the relationship between PA, the decreased risk of depression, anxiety, and stress, as well as improved QoL, sleep and cognitive functions. Nationwide studies offer the strongest empirical data. According to Althumiri et al. (2021) with 8,300 + individuals involved in research, it became clear that following WHO recommendations regarding moderate-intensity physical activity (MIPA) would help reduce the likelihood of MDD and GAD occurrence in particular among men. Although vigorous PA did not demonstrate any benefit in certain cases, even contributing to the increased likelihood of depressive disorders, MIPA was proven as a preventive measure for mental distress (Althumiri et al., 2021). These results highlight the necessity of moderate, sustained activity in the Saudi setting (Table 4).

**Table 4:** Summary of Saudi and regional studies linking physical activity and mental health.

Author/year	Population and sample size	Study design/tool	Key mental health outcomes	Main findings
Althumiri et al. (2021)	National survey, *n* = 8,333 adults	Cross-sectional, PHQ-9, GAD-7	Depression, anxiety	Meeting MIPA guidelines associated with fewer depressive & anxiety symptoms, esp. in men; VIPA not consistently beneficial
Kokandi et al. (2019)	Young adults, *n* = 1,026	Cross-sectional, WHOQOL-BREF, IPAQ	QoL domains	High PA linked with higher scores across all QoL domains; males better in physical health, females higher in social relationships
Abdulrashid et al. (2023)	Adults in Jeddah, *n* = 693	Cross-sectional, GPPAQ, SF-36	HRQoL (PCS, MCS)	Active participants had higher physical and mental HRQoL scores; inactivity linked to poorer outcomes
Zahra et al. (2022)	Adults with/without disability, *n* = 359	Cross-sectional, WHOQOL, PA survey	Psychological QoL	Optimum PA strongly associated with better QoL; sedentary time predicted poor mental health, esp. in disabled
Alnofaiey et al. (2023)	Medical students, *n* = 2,819	Cross-sectional	Depression, stress, academic performance	Higher PA linked with lower depression/stress and better academic performance
Al-Eisa and Al-Sobayel (2012)	Female students, *n* = 105	Cross-sectional	Sleep quality, mood	Regular PA associated with better sleep and mood
Al-Eisa et al. (2014)	Female students, *n* = 76	3-week pedometer intervention	Insomnia, depression, attention	PA improved insomnia, reduced depression, and enhanced attention span
Almaged et al. (2021)	University students, *n* = 500	Cross-sectional, DASS-21	Depression, anxiety, stress	Regular exercisers had lower severe anxiety; no strong associations with depression/stress
Gmmash et al. (2023)	Adolescents, *n* = 27	8-week exercise program	Motivation, well-being	Structured PA improved PA levels, motivation, and psychological well-being
Mahfouz et al. (2023)	School students in Jazan, *n* = 601	Cross-sectional, PedsQL, DASS-21	Depression, anxiety, stress, QoL	PA positively correlated with QoL; reduced mental illness symptoms post-COVID return to school

Elaborating further, Kokandi et al. (2019) established that the greater prevalence of PA was significantly related to all four components of the WHOQOL-BREF, i.e., physical, psychological, social, and environmental health in over 1,000 young adult Saudis (Kokandi et al., 2019). The males demonstrated better physical health, while women had more social relations, implying that men and women benefited from PA regarding their mental health and QoL differently. More research supports these findings. Specifically, Abdulrashid et al. (2023) identified that increased participation in physical activities was related to better physical and psychological HRQoL scores in adults of Jeddah regardless of sociodemographic factors (Abdulrashid et al., 2023). Similarly, Zahra et al. (2022) found that increased peak activity was positively related to psychological QoL in both disabled and healthy individuals, with the latter having more time spent sitting (Zahra et al., 2022).

The university lecturers have provided an interesting view on the relationship between physical activities and mental health. Alnofaiey et al. (2023) found out that increased PA among students in medicine not only helped in reducing stress and depression but also contributed to enhanced academic performance, showing cognitive benefits (Alnofaiey et al., 2023). Al-Eisa and Al-Sobayel (2012) established that PA among female students led to better sleeping habits, decreased cases of insomnia, and reduced depression levels with better focus (Al-Eisa and Al-Sobayel, 2012). The findings are supported by locally collected data in the UAE, which showed that regular exercisers had lower levels of anxiety, with some association with depression and stress (Almaged et al., 2021).

There are many advantages that can be observed from PA interventions among adolescents as well. Gmmash et al. (2023) conducted a study about the effects of eight weeks of aerobic, resistance, and weight-bearing training on Saudi adolescents (Gmmash et al., 2023). Their results demonstrated that, besides an increase in PA, there were positive changes in the situational motivation and psychological well-being, which demonstrates that PA interventions may improve the resilience of the subjects in developmentally sensitive periods. Mahfouz et al. (2023) found that after the COVID-19 pandemic, the greater the PA of Jazan school students was, the better their QoL was and the lower their stress levels were (Mahfouz et al., 2023). In sum, these findings show that PA is a multi-dimensional determinant of mental health in Saudi Arabia which impacts upon mood, cognition, sleep, motivation, and social interactions. Further, it is apparent that moderate physical activity, programs tailored according to gender, and systematic programs at schools/universities can optimize mental health outcomes. These findings are supported by literature from abroad and position PA as a relatively inexpensive option for optimizing mental health outcomes in Saudi Arabian youth.

### 3.5 Barriers to physical activity

Despite the many advantages of physical activity, engagement among young adults in Saudi Arabia is still low, as a result of an interaction among different cultural, social, environmental, and psychological barriers. In particular, gender has emerged as one of the significant factors in determining participation rates. Most studies have indicated that females are under some constraints as a result of cultural beliefs and social conventions that tend to limit their access to sports centers, gender mixing environments, and open recreational facilities. As an example, the findings by Kokandi et al. (2019) indicate that while males had high physical health scores, women registered higher social relationship domain scores, indicating that even though women might get some social benefits from PA, there are still limitations to their general engagement (Kokandi et al., 2019). Additionally, Al-Eisa et al. (2014) found that slight increases in walking activity among females improved insomnia and depression; of course, specific conditions were required (Al-Eisa et al., 2014).

Environmental barriers pose a major challenge as well. Extreme climatic situations, especially very hot weather, make going outdoors undesirable, and the majority of the city infrastructure is unsuitable for walking and having available space outdoors to exercise regularly. Al-Zalabani et al. (2015) and Abdulrashid et al. (2023) pointed out the restriction related to using indoor gyms and shopping malls as major facilities for exercising since one needs money to access the facilities and women-specific places, which are limited (Al-Zalabani et al., 2015; Abdulrashid et al., 2023). The issue applies to young people such as students and early career individuals who have little time to exercise because of their other activities.

Psychosocial factors are equally important. For instance, in their study, Althumiri et al. (2021) proposed that while moderate-intensity physical activities were already known to reduce risks of both depression and anxiety disorders, many people did not meet WHO standards, thus implying some motivational and social issues (Althumiri et al., 2021). Moreover, studies conducted at the university found out that peer support and stress from academics due to pressure, as well as lack of regular routines, decrease adherence to regular physical activity (Alnofaiey et al., 2023; Almaged et al., 2021). Finally, according to Gmmash et al., in young adults, extrinsic support provided through coaching or motivational interventions is extremely important and can help boost motivation (Gmmash et al., 2023).

Additionally, sedentary behavior can be traced back to young adults. Zahra et al. (2022) found that high levels of daily sitting hours beyond five hours led to a significantly poor psychology quality of life among both people with and without disability (Zahra et al., 2022). These results point to a common lifestyle characterized by screen usage for entertainment, education, or work purposes. As found by Mahfouz et al. (2023), the low level of PA among post-COVID-19 restriction school children suffering from mental health problems like stress was linked to poor quality of life in the Jazan region (Mahfouz et al., 2023).

Overall, the examples above provide an understanding that there is inter-locking of barriers to physical activity in Saudi Arabia, where cultural barriers, gender barriers, and lack of access are overlayed with environmental barriers, psychological barriers, and sedentary culture. The solution for overcoming such barriers would involve a multi-level approach that entails investing in infrastructure, creating policies related to gender, support from the community, and programmed efforts aimed at motivating people. Otherwise, individual-level programs may not achieve any success in enhancing the level of PA among youths.

## 4 Public health and policy context

Physical inactivity has been identified as an important public health issue in Saudi Arabia and is considered as a priority in terms of the country’s healthcare reforms under Saudi Vision 2030 (Rakha et al., 2022; Alasqah et al., 2021). One of the core objectives of Vision 2030 includes the promotion of physical activity through sports to increase the number of physically active people in communities to 40 percent by 2030, which currently stands below 20 percent (Rakha et al., 2022). This is one of the goals that Saudi Arabia has set aside in relation to non-communicable diseases but also in relation to the factors affecting mental health, especially among young adults.

There have been many large-scale efforts aimed toward fulfilling this policy. One of the leading steps in integrating community-oriented activities such as walking, mass cycling and outdoor exercises during seasons has been the formation of the Saudi Sports for All Federation. On the other hand, there have been government programs that recognize the positive effects of physical activity on quality of life in terms of recreation, well-being, and social relationships. In addition, there have been urban renewal projects that have focused on building pedestrian facilities, parks, and women’s sporting centers so as to overcome some cultural and environmental constraints that hinder participation in such activities.

This initiative has been spearheaded by universities and educational institutions considering that young people constitute a considerable majority within Saudi Arabia’s demographic profile. Universities have established gymnasiums, sports centers, and well-being facilities for young adults during the last ten years, and many universities have incorporated PA into their comprehensive health initiatives focusing on stress, depression, and lifestyle diseases. Such initiatives are also complemented by efforts from the Ministry of Education which incorporates physical activity in the lives of learners through compulsory sports classes and awareness programs about healthy living. One development which stands out is the growing number of opportunities available for women especially the introduction of physical education classes for girls at school level since 2017 and the licensing of female gyms, which marks a groundbreaking change in policy.

However, although much progress is being made, not enough information is available concerning the effects that these policies have on the mental health of the citizens. From surveillance, there is evidence of steady increase in levels of physical activity; however, it has also been noted that depressive and anxiety disorders continue to be prevalent among college students and young employees. As Althumiri et al. have shown, even with the fulfillment of PA recommendations, other psychosocial factors including cultural influence, stresses from schooling, and lack of physical activity persistently affect mental well-being (Althumiri et al., 2021).

In the future, realization of Saudi Vision 2030’s health goals will depend on multi-sectoral collaboration. Interventions will extend beyond the creation of infrastructure to address behavioral, social, and psychologic barriers through targeted interventions such as peer-led exercise classes, campus health coaching, and web-based health programs that access young adults. Further, the inclusion of mental health monitoring in PA promotion programs will allow for an explanation of the extent to which increased rates of activity are linked with improved psychological outcomes. In this way, Saudi Arabia can build evidence-based interventions that link its ambitious public health reforms to tangible mental health benefits for its young people.

## 5 Research gaps

While there are more studies exploring the connection between physical activity and mental well-being outcome in Saudi Arabia, however, there are significant gaps present. Primarily, most studies are cross-sectional and therefore limit any exploration of the relationship in determining causation of positive outcomes due to physical activities. Moreover, there is a lack of longitudinal studies and intervention studies, including randomized controlled trials. Therefore, there is a lack of research on the effectiveness of physical activity in managing depression, anxiety, or stress in young Saudi individuals.

Finally, there are limited studies concerning young individuals who are not enrolled in university. While much research involves Saudi University students, there has been little focus on the role of physical activity in young adults who become employed or laid off, and who may experience different stressors. The gender differences also remain unknown. While physical activity can be a challenge for women due to cultural restrictions and access to facilities, there is only limited research on the implementation of effective gender-specific intervention trials for encouraging engagement in physical activity.

Last but not least, very little work has been done in assessing national programs such as the Saudi Vision 2030 in terms of their effects on mental well-being. With active policies being developed concerning physical activity, such as building up infrastructures for walking and developing gyms, no concrete evidence-based study has been conducted to assess whether such initiatives result in benefits to mental well-being. This is an area that needs to be addressed in future research.

## 6 Conclusion

As evident from this literature review, physical activity (PA) acts as an important protective factor for psychological well-being among young Saudis, by contributing toward the reduction in depressive and anxious symptoms. Nonetheless, it is important to note that the existing body of literature suggests that such protective associations between physical activity and psychological benefits often face certain sociocultural barriers. Such barriers include the influence of gender-related societal norms, lack of access to PA facilities, and other environmental constraints. While Saudi Vision 2030 provides a model for promoting healthy active lives, the existing literature does not offer any evidence about the effect of such national efforts.
